# Supplementary material for: Effects of the FIFA 11+ and a modified warm-up programme on injury prevention and performance improvement among youth male football players
Source: PLoS One. 2022 Oct 20;17(10):e0275545. doi: 10.1371/journal.pone.0275545 (PMC9584367; doi:10.1371/journal.pone.0275545)
Supplement: Attachment — Submitted filename: response to reviewers.docx [file pone.0275545.s002.docx]

Dear Editor,

*Thank you so much for your great eyes to our work and highlighting those important points. We believe that the points you have raised would greatly enhance the quality of the manuscript. Thus, we tried to follow them in full and revise the manuscript accordingly. More specifically, regarding the data availability policy, we have now uploaded a minimized dataset as per your request. All means and standard deviations have already been mentioned in the manuscript. The changes we made based on your comments are highlighted in red throughout the manuscript.*

More importantly, regarding your following comment ‘’Viewing your abstract, we can see that it was previously published by Springer: "Al Attar, W.S.A., Soomro, N., Pappas, E. et al. How Effective are F-MARC Injury Prevention Programs for Soccer Players? A Systematic Review and Meta-Analysis. Sports Med 46, 205–217 (2016). <https://doi.org/10.1007/s40279-015-0404-x>" *We believe that there must be a mistake simply because any part of this manuscript has not been published up to this point. Further, the link you referred belongs to a systematic review which was first appeared in the literature 6 years ago, whereas this project has only one year old.*

Reviewer #1: Dear authors,
I believe they did a good job and deserve credit for that.
The study is well designed, the introduction is robust, the methods are well explained and the discussion supports the results.
However, the conclusion can and should be improved, and should be presented with practical applications for the coach.

Furthermore, I believe that the limitations should be inserted at the end of the discussion.

*Response: Dear Reviewer, we thank you so much for your positive review and for the positive and useful comments. We revised the conclusion and added recommendations for practitioners and trainers. Additionally, we inserted the limitation at the end of the discussion. The changes we made based on your comments are highlighted in light brown.*

Reviewer #2: The proposed manuscript is very interesting and has a noteworthy objective. Congratulations to the authors.
My suggestions follow below.

*Response: Dear Reviewer, we thank you very much for your positive feedback on our work and for the detailed, supportive and constructive comments. They are very helpful to improve the manuscript’s quality. Therefore, our effort was to integrate the comments throughout the manuscript accurately. The changes based on your comments are highlighted in light blue.*

P.8
Comment 1:

Please rephase the “Background”, specifically “…the latter has potentially reduced compliance and
implementation of the programme”

*Response: Thank you. We revised the background accordingly.*

Comment 2:

Keywords: I suggest not repeating the keyword that already exists in the title to enhance search results.

*Response: Thank you very much. We removed the keywords that are already a part of the title*

Comment 3:

“static stretches” in the warm-up? Or did the authors mean “dynamic stretches”?

*Response: Thank you very much for your great eyes to the important words. According to the reports of our research assistant who was visiting the CG, athletes indeed performed some static stretches within the warm-up phase, with each stretch lasting approximately 12 seconds.*
Comment 4:
Is the discussion the conclusion in the abstract?

*Response: Thank you for your comment. We added a one-sentence conclusion to the abstract.*

P.9
Comment 4:

I suggest rephrasing the sentence “It aims to tackle modifiable injury risk factors such as
neuromuscular control, static and dynamic balance, and the hamstring/quadriceps strength
ratio[8].” as they are not risk factors. They can be considered “key elements of effective injury prevention programmes…”

*Response: Thank you so much. We revised the sentence accordingly.*

Comment 5:

Please rephrase the sentence “The latter, along with barriers such as prolonged duration, concerns regarding some exercises, and lack of a link to football-specific targets[14, 18], has been discussed as a potential reason for low compliance and poor implementation of the programme[14, 19].”

*Response: Thank you so much. We revised the sentence accordingly*.


Comment 6:

Please state the reference for the sentence “Although it is widely accepted that an appropriate warm-up programme should improve performance, more recent studies indicated that the 11+ programme does not improve players' performance acutely but may reduce sprinting and agility compared to a dynamic warm-up programme.”

*Response: Thank you so much. We cited a suitable reference to support the sentence’s content.*

P.10
Comment 7:
In the abstract the authors stated that the CG did not perform injury prevention studies, however in the methods section, the contrary is stated.

*Response: Thank you so much for your great eyes to the manuscript. The phrase ‘’****without any****’’ specific injury prevention exercises… was missing. We rewrote the sentence.*

Comment 7:
Was static stretching truly used?

*Response: Well, it was used and, to my knowledge, is still being used even in some developed countries and on a roughly large scale, unfortunately.*

P.11
Comment 8:
Please add further information of how the instructions to perform the 11+ and the M11+ were carried out.

*Response: Thank you. We would be glad to present further details here and in the manuscript. First, we instructed the trainer and players on how to perform the programmes. Furthermore, we provided the 11+ group with the 11+manual (it was already translated in Persian by IF-MARC, Iran branch of FIFA Medical Assessment and Research Centre) and asked them to familiarize themselves with the 11+. In addition, we provided them with the YouTube link to watch out the 11+ exercises. For the M11+, we provided the trainers and the players with a series of photo slides including all exercises embedded in the programme. Meanwhile, to assure accuracy of the application of the 11+, a research assistant was responsible for visiting the training sessions of the M11+ and F11+ groups and checking the implementation of the warm-up modalities.*

Comment 9:
Data collection and supervision: How where the assistants “blinding each other”?

*Response: Thank you so much for highlighting these important points. We applied that approach to minimize any potential risk of bias during data collection. Three research assistants were responsible for their subsequent groups while having no idea about the study aims, the interventions, occurred injuries of the other groups, etc. They were only in touch with the research group leader and remained anonymous across the project.*

Comment 10:

Why was the “M. iliopsoas strengthening” and the “single-leg standing with a heel-to-toe movement cycle” selected? Can the authors support these choices with evidence? It would be great as the M11+ group showed few injuries.

*Response: Thank you very much for your detailed and beneficial comments. We are pleased to briefly explain it for your perusal:*

*The idea of strengthening M. iliopsoas muscles and single-leg standing with a heel-to-toe movement cycle and also modifying some core exercises comes from Janda’s approach, namely, the muscular chain, which is also known as upper/lower cross syndromes, as well as Shirley Sahrmann’s approach:*

*Izraelski, J., Assessment and treatment of muscle imbalance: The Janda approach. The Journal of the Canadian Chiropractic Association, 2012.* ***56****(2): p. 158.*

*Sahrmann, S., D.C. Azevedo, and L. Van Dillen, Diagnosis and treatment of movement system impairment syndromes. Brazilian journal of physical therapy, 2017.* ***21****(6): p. 391-399.*

P.12
Comment 11:
A table with Injury incidence/1000 h and rate ratios for each group would enhance the manuscript.

*Response: Thank you for your suggestion. We appreciate your support for enhancing the manuscript and considering them all. In this case, however, as we already have three tables in the manuscript, we taught that having another table could be too much. Therefore, we just added a row to table two, including IIC rates.*

Comment 12:
Further detail on injuries would also be of great interest for the study: (Fuller et al, 2006).

*Response: Thank you. We added additional information.*
p.13
Comment 13:
The second manuscript from (Lopes et al.) is not in the references section. In the sentence extracted from other manuscripts as the numbered references are of other studies?

*Response: Thank you so much for highlighting that mistake. Following your comment, we went through the reference list and found some errors by the meaning of false matching the numbers and references. Thus, all references were updated and matched accurately.*
p.14
Comment 14:
“Neither F11+ nor M11+ could significantly reduce the risk of groin and hip injuries.”
Can these stats be shown in a table?

Response: Thank you for your attention. Given the few injuries that occurred in the hip and groin, we considered them and reported all as thigh injuries.

Comment 15:
The paragraph below is very important, however the message is not clear. Please rephrase.

“Neither F11+ nor M11+ could significantly reduce the risk of groin and hip injuries. In addition to the reasons mentioned above, they could not mitigate hip and groin injuries since they provided no specific exercise for groin muscles. This appears to be a noticeable gap, as up to 12% of subelite football injuries occur in the groin region [41]. Wahlan et al. suggested that using Copenhagen adductor exercises may fill this gap and would be fit for inclusion in the FIFA11+ programme, as it requires no specific equipment and has three levels of difficulties [41]. The better efficacy of the M11+ in preventing hip injuries, however, might be due to increasing the volume of hip dynamic stretch exercise, namely, hip-in, in addition to manipulating how core area muscles are being trained.

*Response: Thank you for your comment. We revised the paragraph and hope that the readers now can clearly follow the argumentation and get the message.*

P.15
Comment 16:
Limitations
In fact the study did not recruit the expected number of teams to manage a randomized sample. It is not clear that the authors did recognize this limitation.

*Response: Thank you. We revised the limitation and pointed out the lack of a recruiting sample in addition to the short monitoring period.*

Comment 17:
Was the trial registered in a clinical trials platform?

*Response: Thank you. It was not registered*
