# Supplementary material for: Effects of the FIFA 11+ and a modified warm-up programme on injury prevention and performance improvement among youth male football players
Source: PLoS One. 2022 Oct 20;17(10):e0275545. doi: 10.1371/journal.pone.0275545 (PMC9584367; doi:10.1371/journal.pone.0275545)
Supplement: Attachment — Submitted filename: Response to the reviewers second round.docx [file pone.0275545.s003.docx]

Dear respected editor/ reviewers

On behalf of our research group, I do thank you so much again for your positive reviews and commentary on our work. We do believe that the comments you made have widely improved the quality of our paper, so we highly appreciate your efforts. Just to reflect well to the last comment provided by the second reviewer which is a kind of misunderstanding/miscommunication, I do refer to the first revision round where I clearly stated that based on the research assistant, the Control Group (CG) used some static stretches as part of their routine warm up before main training. So, there was no any kind of static stretches neither in 11+ nor in the modified 11+ and as you accurately mentioned, it has been scientifically proven that static stretches must not be used for warming up.

To eliminate such misunderstanding, bellow you’ll find the original comments and the responses from the first revision round:

Comment 3:

**“static stretches”** in the warm-up? Or did the authors mean “dynamic stretches”?

*Response: Thank you very much for your great eyes to the important words. According to the reports of our research assistant who was* ***visiting the CG****, athletes indeed performed some static stretches within the warm-up phase, with each stretch lasting approximately 12 seconds.*

Comment 7:
Was **static stretching** truly used?

*Response: Well, it was used and, to my knowledge, is still being used even in some developed countries and on a roughly large scale, unfortunately.*

Given that there is no other comments to be reflected, I assume that this clarification suffices and therefore, we did not provide any changes on the manuscript.
